# Lifelong Ulk1-Mediated Autophagy Deficiency in Muscle Induces Mitochondrial Dysfunction and Contractile Weakness

**DOI:** 10.3390/ijms22041937

**Published:** 2021-02-16

**Authors:** Anna S. Nichenko, Jacob R. Sorensen, W. Michael Southern, Anita E. Qualls, Albino G. Schifino, Jennifer McFaline-Figueroa, Jamie E. Blum, Kayvan F. Tehrani, Hang Yin, Luke J. Mortensen, Anna E. Thalacker-Mercer, Sarah M. Greising, Jarrod A. Call

**Affiliations:** 1 Department of Kinesiology, University of Georgia, Athens, GA 30602, USA; asnichenko@vt.edu (A.S.N.); wsouther@umn.edu (W.M.S.); ags27911@uga.edu (A.G.S.); jennifer.mcfalinefigueroa@uga.edu (J.M.-F.); 2 Regenerative Bioscience Center, University of Georgia, Athens, GA 30602, USA; anita.qualls@ucsf.edu (A.E.Q.); kayvan.tehrani@uga.edu (K.F.T.); luke.mortensen@uga.edu (L.J.M.); 3 School of Kinesiology, University of Minnesota, Minneapolis, MN 55455, USA; sorensej@umn.edu (J.R.S.); grei0064@umn.edu (S.M.G.); 4 Division of Nutrition Science, Cornell University, Ithaca, NY 14850, USA; jeb462@cornell.edu (J.E.B.); athalack@uab.edu (A.E.T.-M.); 5 Center for Molecular Medicine, University of Georgia, Athens, GA 30602, USA; hyin@uga.edu; 6 Department of Cell, Developmental and Integrative Biology, University of Alabama, Birmingham, AL 35294, USA

**Keywords:** aging, mitophagy, autophagy flux, neuromuscular junction, sarcopenia

## Abstract

The accumulation of damaged mitochondria due to insufficient autophagy has been implicated in the pathophysiology of skeletal muscle aging. Ulk1 is an autophagy-related kinase that initiates autophagosome assembly and may also play a role in autophagosome degradation (i.e., autophagy flux), but the contribution of Ulk1 to healthy muscle aging is unclear. Therefore, the purpose of this study was to investigate the role of Ulk1-mediated autophagy in skeletal muscle aging. At age 22 months (80% survival rate), muscle contractile and metabolic function were assessed using electrophysiology in muscle-specific Ulk1 knockout mice (MKO) and their littermate controls (LM). Specific peak-isometric torque of the ankle dorsiflexors (normalized by tibialis anterior muscle cross-sectional area) and specific force of the fast-twitch extensor digitorum longus muscles was reduced in MKO mice compared to LM mice (*p* < 0.03). Permeabilized muscle fibers from MKO mice had greater mitochondrial content, yet lower mitochondrial oxygen consumption and greater reactive oxygen species production compared to fibers from LM mice (*p* ≤ 0.04). Alterations in neuromuscular junction innervation patterns as well as changes to autophagosome assembly and flux were explored as possible contributors to the pathological features in Ulk1 deficiency. Of primary interest, we found that Ulk1 phosphorylation (activation) to total Ulk1 protein content was reduced in older muscles compared to young muscles from both human and mouse, which may contribute to decreased autophagy flux and an accumulation of dysfunctional mitochondria. Results from this study support the role of Ulk1-mediated autophagy in aging skeletal muscle, reflecting Ulk1′s dual role in maintaining mitochondrial integrity through autophagosome assembly and degradation.

## 1. Introduction

Autophagy is an evolutionarily conserved cellular process for degrading damaged and dysfunctional proteins and organelles and has been strongly associated with the age-related reduction in skeletal muscle mass and function [1,2,3]. Studies investigating autophagy in older adults and rodents report less basal autophagy signaling, as well as less mitochondrial-specific autophagy (i.e., mitophagy) signaling with age [4,5,6,7,8]. Moreover, autophagy-related protein knockout models indicate an exacerbated aging phenotype that supports an important role for autophagy in healthy skeletal muscle aging [7,9,10,11,12]. For example, Carnio et al. discovered geriatric mice with deficient ATG7, an autophagy-related protein important for autophagosome assembly, have weaker muscles, greater levels of muscle fiber atrophy and dysfunctional neuromuscular junctions (NMJs) compared to age-matched controls [7]. Autophagy is a dynamic process that can be altered not only by changing the number of autophagosomes assembled but also by changing the rate of autophagosome degradation (i.e., autophagy flux). In addition to changes in autophagosome assembly, there is compelling research to suggest that autophagic flux is altered with age [10,11,12]. Therefore, it appears that autophagy is inextricably linked with aging and changes in both autophagosome number and flux may lead to an accumulation of damaged organelles and proteins, which contribute to the progression of skeletal muscle aging.

The accumulation of damaged and dysfunctional mitochondria with age is thought to contribute in part to sarcopenia, due to metabolic insufficiency and a greater production of reactive oxygen species (ROS) [13,14,15,16]. Damaged and ROS-producing mitochondria can be degraded through mitophagy, which is regulated through Unc-51 like autophagy activating kinase, Ulk1 [17,18,19]. Ulk1 has long been thought of as an autophagy-initiating protein that controls autophagosome assembly, but recent mechanistic investigations in yeast suggest that Ulk1 may also be involved in the signaling for autophagosome-lysosome fusion that leads to autophagosome degradation [20]. Thus, Ulk1 appears to play a mechanistic role in autophagosome assembly and flux. Yet the extent to which Ulk1 influences sarcopenia is unclear.

The purpose of this study was to investigate the effects of lifelong insufficient Ulk1-mediated autophagy on aging skeletal muscle phenotypes to better understand the contribution of reduced autophagy to sarcopenia. We hypothesized that lifelong Ulk1-defiency will exacerbate sarcopenia as indicated by a worsening of skeletal muscle contractile and metabolic function. Herein, we utilize highly sensitive electrophysiological techniques to assess muscle contractile and metabolic function in aged mice with genetic deletion of Ulk1 and their littermate controls. We also assess changes of autophagy signaling and flux in young LM and Ulk1 knockout mice to gain insight into potential mechanisms related to the role of autophagy in the development of sarcopenia.

## 2. Results

**Figure 1:**
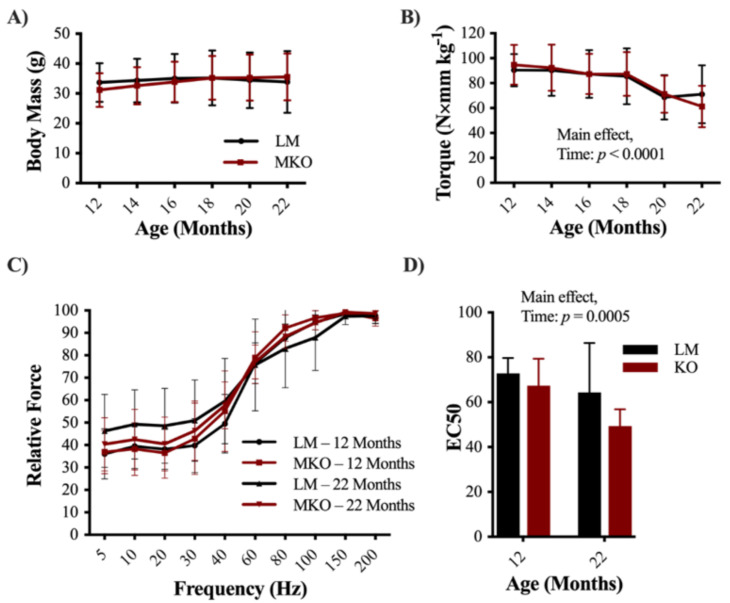
Decline in skeletal muscle function with age (**A**) Average longitudinal body mass measurements of Ulk1 muscle-specific Ulk1 knockout mice (MKO) mice and littermate controls (LM) controls starting at 12 months of age through 22 months. (**B**) Average longitudinal in vivo muscle peak torque measurements for 12–22 months of age. (**C**) Relative force frequency of LM and MKO mice at 12 and 22 months of age. (**D**) EC50 calculated from longitudinal force frequency measurements at 12 and 22 months of age. All data are presented as mean ± SD *n* = 10 mice. Main effects are listed where observed.

Ulk1 autophagy-deficient mice (MKO) at 22 months of age were used to interrogate the importance of Ulk1 protein signaling in otherwise healthy skeletal muscle aging. Body mass did not differ between littermate wildtype (LM) age-matched controls and MKO mice or change significantly with time during the longitudinal portion of this study (12–22 months; Figure 1A, *p* = 0.418). We also investigated muscle masses after sacrifice and did not find any differences between genotypes in TA, EDL, or SOL muscle masses (Figure S1, *p* ≥ 0.07). However, gastrocnemius and heart muscle mass were significantly lower in MKO mice when normalized to body mass (Figure S1, *p* ≤ 0.04). Over the 12-month longitudinal period there was a 40% loss of peak-isometric dorsiflexion torque independent of genotype (Figure 1B, *p* < 0.001). There was no significant interaction in force frequency curve shifts, however there was a main effect of both age and genotype (Figure 1C, Main effect: Age and Genotype *p* = 0.037, *p* = 0.014, respectfully). To further decipher this data, curve fits were applied to torque-frequency data to determine the frequency at which 50% of peak-isometric torque was achieved (EC50), and whether EC50 changed with age and/or genotype. The EC50 frequency decreased from 70 to 52 Hz with age, independent of genotype (Figure 1D, Main effect: Age *p* < 0.001). Torque-time tracings were also analyzed for changes in contractile properties with age and genotype, but there were no significant interactions or main effects (e.g., twitch half-relaxation time; Table S1, *p* ≥ 0.489).

**Figure 2:**
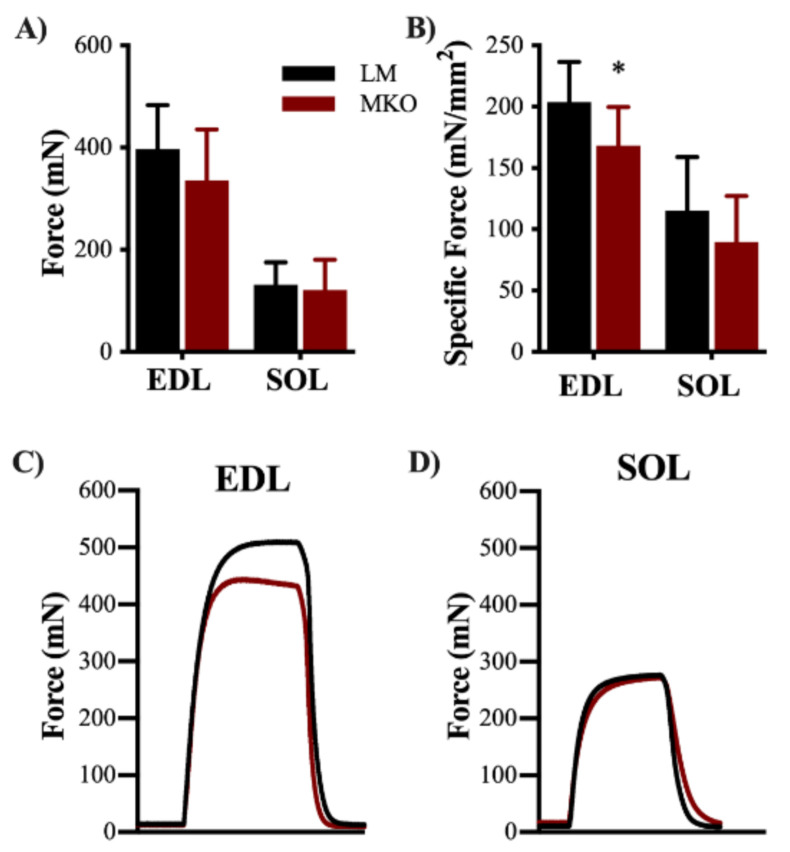
Ulk1 deficiency predominately affects fast-twitch muscle fibers strength. (**A**) Average in vitro maximum contraction force from both extensor digitorum longus (EDL) and soleus (SOL) muscles (*p* > 0.05) (**B**) Average specific force from both EDL and SOL muscles (*p* = 0.0277, and *p* = 0.2371, respectfully). Representative torque tracing from (**C**) EDL and (**D**) SOL muscles. All data are presented as mean ± SD *n* = 10 mice. * = Significantly different from LM.

In vitro contractility of the EDL and SOL muscles was assessed to determine changes in slow- versus fast-twitch muscle properties with lifelong Ulk1 deficiency. There was no difference in EDL muscle peak-isometric force (Figure 2A, *p* = 0.175), however, when accounting for physiological CSA, EDL muscle specific force production was 17% less in the MKO mice compared to the LM (Figure 2B, *p* = 0.028). No difference was detected between genotypes for either SOL muscle peak-isometric force or specific force (Figure 2A,B, *p* = 0.237). Force-time tracings were also analyzed for changes in contractile properties with age and genotype, but there were no significant interactions or main effects (e.g., twitch half-relaxation time) (Table S2, *p* ≥ 0.068).

**Figure 3:**
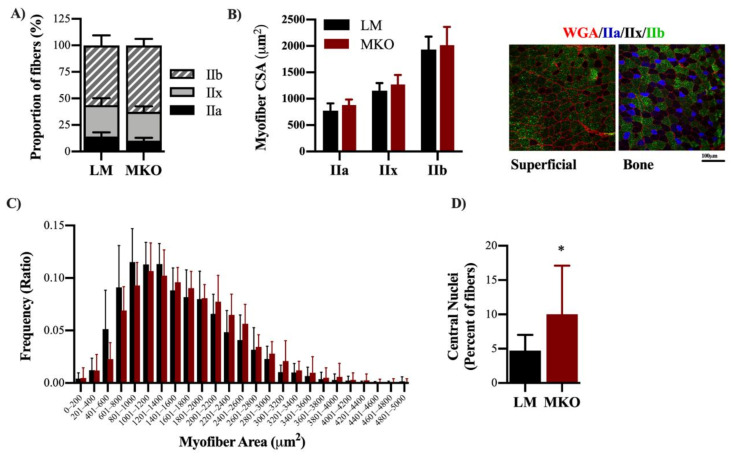
Ulk1 deficiency throughout life leads to altered muscle fibers anatomy. (**A**) Distribution of fiber types within TA muscle sections analyzed and representative images of fiber type staining. (**B**) CSA of different fiber types and representative images of fiber type staining. (**C**) Frequency distributions of fiber CSAs from TA muscles. (*p* = 0.0035) (**D**) Percent of centrally located nuclei (*p* = 0.0487) All data are presented as mean ± SD. * = significantly different from LM.

The TA muscle was selected for analysis of fiber number, myosin heavy chain (MyHC) fiber type distribution, and CSA because it is the primary muscle involved in ankle dorsiflexion torque and because of its heterogeneous fast-twitch fiber type distribution. There was no difference in total fiber number between genotypes (2342 ± 498 vs. 2036 ± 678, LM and MKO, respectively, *p* = 0.264). There were no differences in the proportion of type IIa, IIb, or IIx fibers between genotypes (Figure 3A, *p* ≥ 0.196). There were differences in fiber type-specific CSA such that IIa fibers were the smallest and IIb were the largest (Figure 3B, *p* ≤ 01), yet no difference between genotypes was observed (*p* = 0.963). Intriguingly, the distribution of overall fiber CSA independent of fiber type was shifted rightward so that the mean CSA of all fibers was 10% greater in the TA muscle of MKO compared to LM (1539 ± 215 vs. 1692 ± 248 µm^2^, LM and MKO, respectively) which is indicative of larger muscle fibers overall in MKO compared to LM (Figure 3C, *p* = 0.004). The TA muscle contributes greater than 80% to peak-isometric torque of the ankle dorsiflexors, so we retroactively analyzed peak-isometric torque normalized by the TA muscle CSA. Specific peak-isometric torque was 15% less in MKO mice compared to LM controls (Figure S2, *p* = 0.008).

**Figure 4:**
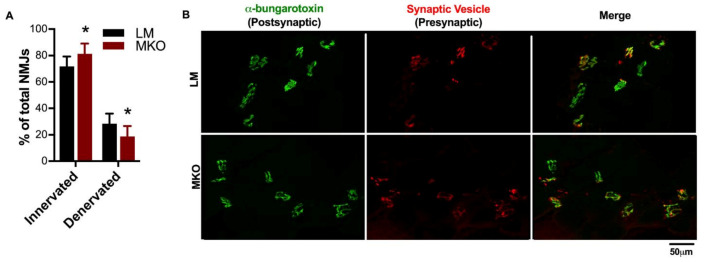
Neuromuscular junction integrity changes with lifelong Ulk1 deficiency. (**A**) Average number of innervated and denervated neuromuscular junctions (NMJs) in the diaphragm of MKO and LM mice (*p* = 0.0227). Data are presented as mean ± SD. * = significantly different from LM. (**B**) Representative images of the pre- (red) and post-synaptic (green) terminals of the NMJs from both MKO and LM diaphragm muscle sections. The merged image depicts both innervated (yellow) and denervated (green) NMJs. Innervation was defined as the overlap of pre- and post-synaptic structures, while the complete or partial absence of the presynaptic terminal classified the NMJ as denervated.

During the blinded fiber-type and CSA analysis, a number of samples with a high percentage of centrally-located nuclei were noted. Centrally-located nuclei in the absence of muscle injury can be indicative of muscle fiber death and natural turnover with age. In agreement with a previous report involving muscle-specific Atg7 knockouts [7], there was a greater percentage of centrally-located nuclei in autophagy deficient MKO muscles compared to LM (Figure 3D, *p* = 0.049). Previous aging research and studies involving autophagy-deficient models have revealed changes in NMJ integrity [7,21]. The diaphragm muscle was selected to determine if Ulk1 deficiency influenced NMJs because the diaphragm muscle displays all fiber types and is necessary for life, providing evaluation of a muscle in-between the predominantly slow- and fast-twitch limb muscles. Innervation was determined by evaluating the presynaptic [synaptic vesicle and neurofilament (red)] and postsynaptic [α-bungarotoxin (green)] structures of the NMJ. The NMJ is a specialized chemical synapse between the motor neuron and muscle fibers that facilitates the transmission of an action potential, causing muscle contraction. Innervation was defined as the significant overlap of pre- and post-synaptic structures (i.e., yellow merged images), while the complete or partial absence of the overlap classified the NMJ as denervated (i.e., mostly green merged images). The MKO mice had a greater proportion of innervated fibers and less denervated fibers compared to age-matched LMs (Figure 4A,B, *p* = 0.023). The frequency of denervation in the age-matched LM is comparable to that previously reported [21], and follows a period of rapid denervation between middle and old age. We suspect our evaluation comes at a time in which many fibers have been lost to lack of innervation, i.e., there was survivor bias.

**Figure 5:**
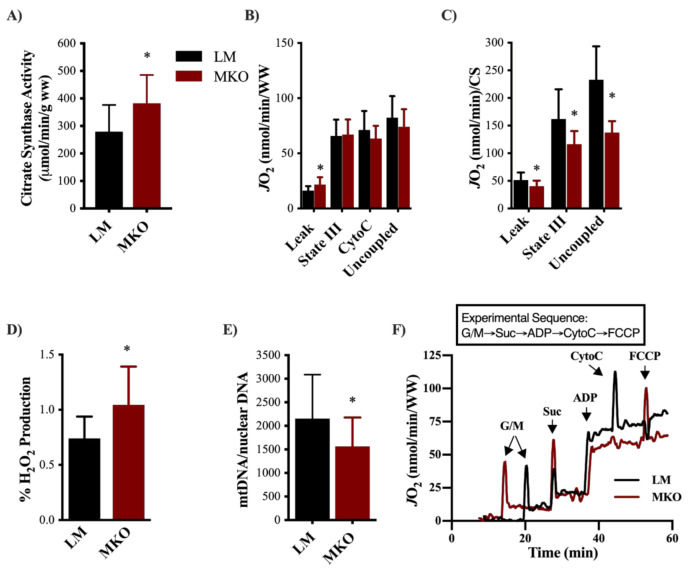
Lifelong Ulk1 deficiency results in an accumulation of dysfunctional mitochondria. (**A**) Mitochondrial content assessed through citrate synthase enzyme kinetic rates (*p* = 0.0443). (**B**) Mitochondrial oxygen consumption during Leak, State III, cytochrome C (to check for intact mitochondrial membrane), and uncoupled respiration normalized to wet tissue weight. (*p* = 0.026) (**C**) Mitochondrial oxygen consumption during Leak, State III, and uncoupled respiration normalized to citrate synthase activity. (*p* = 0.041, *p* = 0.002, *p* < 0.001, respectively) (**D**) Percent ROS production normalized to oxygen consumption during leak respiration to account for differences in oxygen flux (*p* = 0.0428). (**E**) Mitochondrial DNA content normalized to nuclear DNA content (*p* = 0.0309). (**F**) Representative oxygen flux measurements during a mitochondrial respiration protocol (O2k Oroboros). All data are presented as mean ± SD. * = significantly different from LM.

**Figure 6:**
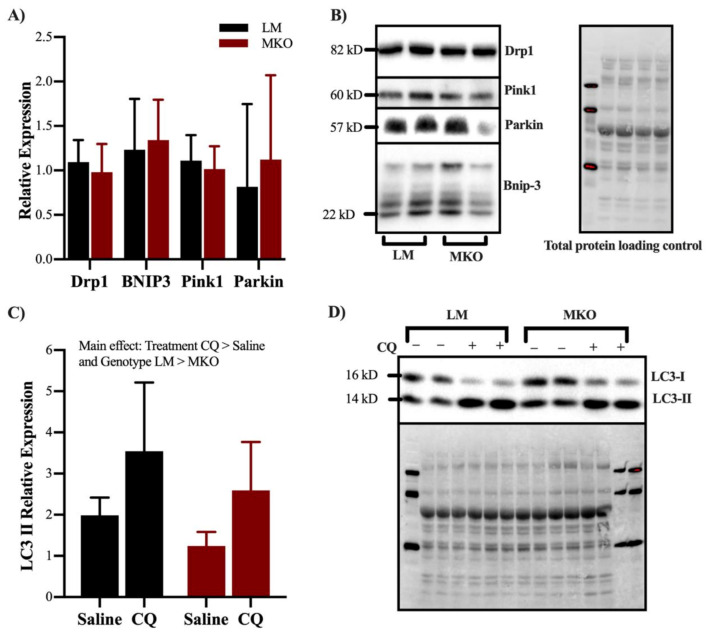
Effect of Ulk1 deficiency on markers of mitochondrial remodeling. (**A**) Relative expression of mitophagy related proteins DRP1, BNIP3, Pink1, and Parkin (*p* > 0.05), (**B**) Representative immunoblot panel (**C**) Relative expression of LC3II in middle aged mice treated with Choloroquine or saline as a control (Main effect of Treatment and Genotype *p* = 0.0007 and *p* = 0.0341, respectfully). (**D**) Representative immunoblots of LC3. All data are presented as mean ± SD.

Ulk1-mediated autophagy is one of the main degradative pathways for skeletal muscle mitochondria, therefore we decided to investigate mitochondrial content, function, and ROS production after lifelong Ulk1 deficiency. Mitochondrial content (i.e., citrate synthase activity) of the gastrocnemius muscle was 37% greater in the MKO muscle fibers compared to LM (Figure 5A, *p* = 0.044). Leak respiration (malate/glutamate/succinate) of permeabilized muscle fibers as a function of mass (grams) was 34% greater in MKO compared to LM (Figure 5B, *p* = 0.026). When respiration rates were normalized to mitochondrial content, fibers from MKO mice had 28% less State III (malate/glutamate/succinate/saturated ADP) and 41% less uncoupled (FCCP-uncoupled) respiration compared to LM controls (Figure 5C, *p* < 0.02). MKO permeabilized muscle fibers also produced more ROS per unit of oxygen flux compared to LM permeabilized muscle fibers (Figure 5D, *p* = 0.043). To determine the extent to which Ulk1 deficiency and greater ROS production damaged mitochondria, the amplification of mtDNA by PCR was determined as poor amplification reflects poor mtDNA integrity. mtDNA amplification was significantly lower in MKO mice compared to LM controls (Figure 5E, *p* = 0.031). To gain insight into potential causes of an accumulation of dysfunctional mitochondrial with age in MKO mice, we explored markers of mitochondrial remodeling and autophagy flux in a cohort of middle-aged MKO and LM mice (see Experimental Design for explanation on the use of middle aged mice). In a panel of proteins typically indicative of mitochondrial remodeling (DRP1, BNIP3, Pink1 and Parkin), there were no differences between genotypes in middle-aged mice (Figure 6A,B, *p* > 0.05). Basal autophagy flux was assessed by an acute treatment with the lysosomal inhibitor Chloroquine (CQ). CQ treatment resulted in greater accumulation of LC3II protein content independent of genotype (*p* = 0.0007), and MKO mice had less LC3II protein content (Figure 6C,D, *p* = 0.0341). These data suggested that autophagosome degradation is different in MKO mice but are inconclusive on the extent to which autophagy flux is altered specifically because there lacked a stimulus to induce autophagy flux (e.g., fasting).

**Figure 7:**
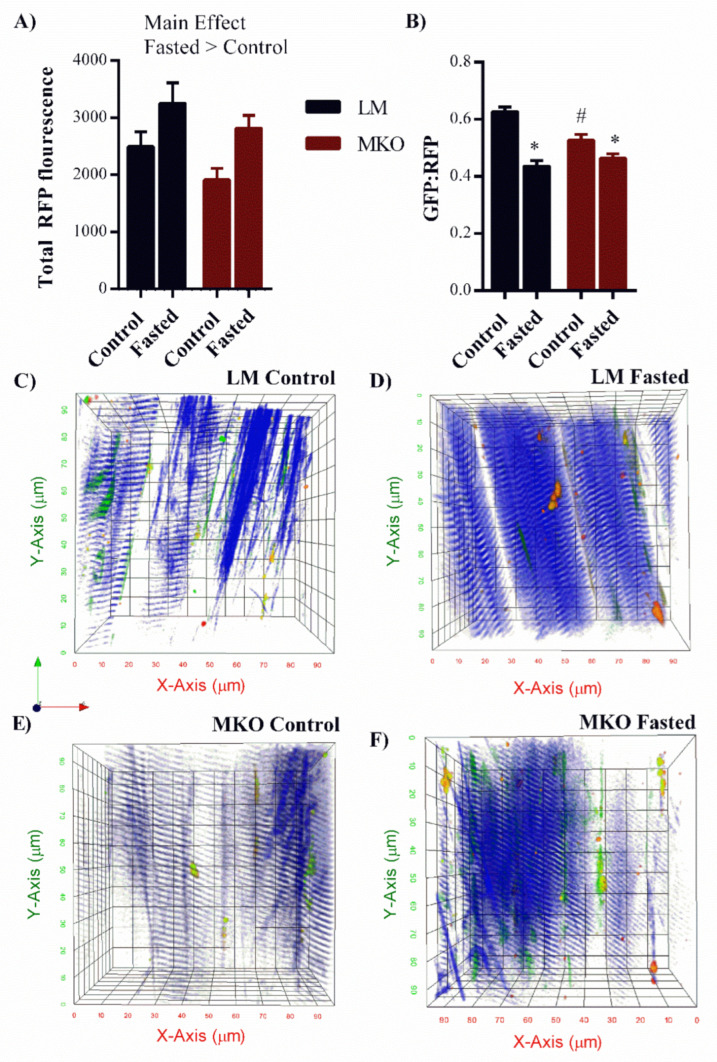
Altered autophagy flux response to fasting in young Ulk1 MKO muscles. (**A**) Quantification of total red fluorescence intensity in fasted and control MKO and LM muscles (Main effect of fasting, *p* = 0.003). (**B**) Green:Red fluorescent protein ratio after fasting compared to control levels of autophagosome degradation in Ulk1 MKO and LM muscles, Significant interaction (*p* = 0.006) All data are presented as mean ± SEM * = significantly different from both control groups, # = significantly different from LM control. Representative two photon z-stack images taken from the TA muscle of (**C**) LM control, (**D**) LM fasted, (**E**) MKO Control, (**F**) MKO fasted mice. Green = LC3II incorporated into autophagosome membrane, Red = LC3II in cytosol, Blue = second harmonic generation labeling skeletal muscle contractile proteins for identifying muscle fiber location.

To better assess autophagy flux, basal and fasting autophagy flux was assessed using multi-photon microscopy and transfection with the GFP-LC3-RFP-LC3 plasmid (Figure 7A–F). Following successful transfection, RFP and GFP-LC3 proteins are synthesized in tandem where the RFP-LC3 externalizes outside the autophagosome and serves as a baseline fluorescent control and the GFP-LC3 is internalized within the autophagosome and its fluorescent signal is extinguished upon autophagosome-lysosome fusion and subsequent degradation. The RFP signal is used to estimate total autophagosomes and a decrease in the fluorescent GFP:RFP ratio reflects greater autophagy flux. Second harmonic generation (blue) generates intrinsic contrast from the organized actin/myosin bands and so was used to visualize the contractile elements orientation of the muscle fiber. The RFP fluorescence was 30% greater in fasted muscle compared to basal muscle independent of genotype suggesting greater autophagosome assembly with the fasting protocol (Figure 7A, Main effect: Fasting *p* = 0.003). Compared to basal autophagy flux, fasting resulted in a 29% reduction in the GFP:RFP ratio in LM mice (Figure 7B, *p* < 0.001). This is represented as a “red shift” in in LM-Fasted compared to LM-Fed (Figure 7C,D) as relative green fluorescence lessens. This response was partially blunted in MKO mice, which only experienced a 15% reduction in the GFP:RFP ratio in response to fasting (Figure 7B, *p* < 0.001) and fibers had a noticeably greener and/or yellow pattern (Figure 7D–F) (LM-Fasted vs. MKO-Fasted). These data suggest that MKO mice have an impaired ability to stimulate autophagy flux in response to fasting and that could have larger implications on the ability of MKO mice to upregulate flux in response to other stimuli throughout life (e.g., exercise, muscle damage).

**Figure 8:**
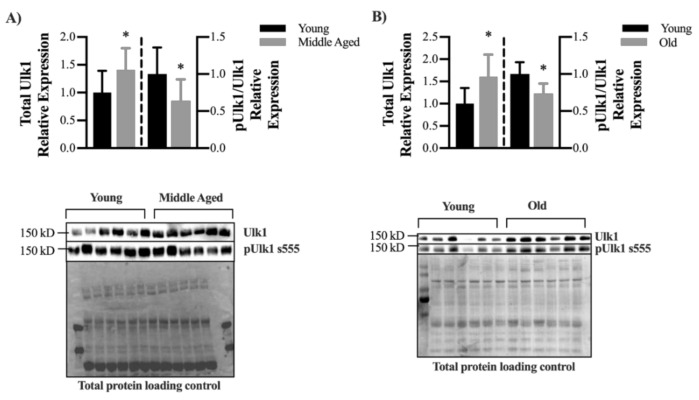
Decline in Ulk1 phosphorylation with age (**A**) Relative expression of total Ulk1 and pUlk1 normalized to Ulk1 in young (4 months) and middle-aged (16 months) mice and representative immunoblot images. Mean ± SD *n* = 7 mice. * = significantly different from young. (**B**) Relative expression of total Ulk1 and pUlk1 normalized to Ulk1 in young (20–40 y) and old (60–80 y) human muscle biopsy samples and representative immunoblot images. Mean ± SD *n* = 6 samples. * = significantly different from young. Bars on the left of the dashed lined correspond to the left *y*-axis, and bars on the right of the dashed line correspond to the right *y*-axis.

Finally, age-related declines in autophagy-related proteins LC3 (MAP1LC3A) and ATG7 have previously been reported, and these declines are thought to contribute to sarcopenia [7]. In order to better understanding the influence of aging on Ulk1 protein content and activation, we assessed total Ulk1 protein content and phosphorylated (s555) Ulk1 protein content in young and middle-aged mouse muscle fibers as well as in old and young human muscle fibers. Total Ulk1 was increased 41% in middle aged mice compared to young mice however the ratio of active to total Ulk1 was 36% less in 16-month compared to 4-month LM mice (Figure 8A, *p* = 0.0239 and *p* = 0.017, respectively). Similarly, although there was significantly greater total Ulk1 protein content in old (60–80 years) compared to young (20–40 years) human muscle (+ 60%, *p* = 0.049), the ratio of active to total Ulk1 was 26% less in old muscles versus young muscles (Figure 8B, *p* = 0.015).

## 3. Discussion

A decline of mitochondrial function throughout life is a contributor to many aging phenotypes [2,3,10,12], and this decline is associated with an increase in ROS production [22]. Herein, we observed a greater number of mitochondria which consumed less oxygen and produced more ROS in the MKO mice compared to LM controls (Figure 5). Though there are several sources of ROS production within skeletal muscles (e.g., xanthine oxidase, NAD(P)H oxidase), a majority of ROS is the by-product of electrons leaking from complexes I and III of the electron transport chain creating superoxide anion [23]. Superoxide anion quickly dismutates to form the more stable, membrane permeable H_2_O_2_ that we detected in higher quantities in MKO mice compared to LM (Figure 5). mtDNA, which helps maintain the integrity of the electron transport chain, is susceptible to ROS-induced mutations due to its proximity to ROS production within the mitochondria and its lack of DNA-repair processes [24,25]. While mtDNA mutations in our model are unclear, we did detect less mtDNA amplifcation in MKO mice (Figure 5), a marker of mtDNA damage. Age-related declines in skeletal muscle mtDNA amplification have been reported in humans [26] and can associate with ROS production and mitochondrial dysfunction [27]. While low (i.e., physiological) levels of ROS production can act as a positive influence on muscle function [28], sustained and/or excess levels (i.e., pathological) are known to contribute to skeletal muscle dysfunction with age [29]. Previously, we have not detected differences in mitochondrial function in healthy skeletal muscles from MKO mice and LM [30], leading us to speculate that the aging phenotype herein resulted from improper maintenance of the mitochondrial network with time.

Several cellular processes are responsible for maintaining the quality of the mitochondrial network during pathological ROS production, such as antioxidant defense enzymes, fission proteins to remove damaged mitochondria, and autophagy proteins to isolate and degrade damaged mitochondria. Mitochondrial fission, a precursor for autophagy, is facilitated by Drp1 forming around mitochondria and severing damaged portions from the network [31]. The severed, dysfunctional mitochondrial unit is next targeted for selective degradation by full-length PINK1 protein expression and then Parkin recruitment to the outer mitochondrial membrane to initiate compartmentalization by an autophagosome [32]. Herein, the expression of these mitochondrial network remodeling proteins appears to be independent of Ulk1 (Figure 6). This finding agrees with reports that Ulk1 and Drp1 are activated in concert but under the control of different upstream signaling cascades [19]. Therefore, the accumulation of dysfunctional mitochondria in MKO mice is likely not due to a failure of damaged mitochondrial separation and targeting, but more an inability to sufficiently degrade them in a timely manner (e.g., limited Ulk1-mediated autophagy flux).

Ulk1 is one of the few autophagy-related kinases and its various roles in autophagy signaling and cellular maintenance are still being elucidated. Autophagy broadly involves the (1) induction, nucleation, and expansion of the autophagosome, (2) cargo selection and compartmentalization, and (3) degradation [33]. Ulk1 is the mammalian homolog to Atg1 that has been shown to be necessary and sufficient for autophagy-induced lifespan extension in yeast and Drosophila [34,35]. In skeletal muscles, Ulk1 appears to play a unique role in mitophagy signaling as it is sensitive to shifts in energy states and is post-translationally modified by energy sensing proteins like AMPK [17,36]. AMPK phosphorylates Ulk1 at s555, prompting Ulk1 to co-localize to the mitochondria which is responsible for inducing mitophagy, as is the case in response to exercise [19]. Specifically, AMPK-induced Ulk1 co-localization recruits’ autophagy-related machinery and lysosomes to the mitochondria to complete autophagic degradation [19,20]. Not surprisingly, aged AMPK-deficient mice have weaker muscles with enlarged and damaged mitochondria (reduced mtDNA and increased ROS) similar to the data presented herein with Ulk1 MKO (Figure 5) [9]. Overall, this supports the AMPK-Ulk1 signaling cascade for the degradation of damaged mitochondria through mitophagy is important with age. Post-translational modifications and kinase activities of Ulk1 were not within the scope of this study. However, it is critical to evaluate the cellular signaling linking Ulk1 to cellular maintenance and autophagy within skeletal muscle.

Autophagy is a dynamic process and active autophagosome degradation must be considered when assessing overall autophagic function [37]. We have previously shown that autophagy signaling is greatly upregulated in response to muscle injury, but autophagic flux does not increase to the same extent which results in an autophagosome clearance bottleneck [30,38]. The extent to which aging influences the autophagosome assembly and flux relationship is unclear. However, recent research has expanded the role of Ulk1 associating it with regulating autophagy flux [20]. Specifically, Wang et al. reported that in yeast, Atg1 (a Ulk1 homologue) kinase activity regulates the tethering of autophagosomal and lysosomal SNARE proteins, which leads to autophagolysosome fusion and subsequent degradation [20]. Considering this newly discovered role of Ulk1 in autophagy flux regulation combined with our mouse and human data showing lessened Ulk1 activation with age (Figure 8) it is worth considering how Ulk1′s dual role in autophagy may be influencing muscle quality with age. Furthermore, we found herein that young MKO mice were not able to increase flux in response to a fasting stimulus to the same extent as LM mice (Figure 7). This may represent a potential mechanism leading to an accumulation of damaged mitochondria with age because the MKO mice are unable to adequately upregulate degradation in response to certain stress stimulus.

Similar findings of accumulated damaged mitochondria have been found in other aging autophagy knockout models [7,9]. Interestingly, our data revealed predominately fast-twitch muscle, comprised of type IIa/IIb fibers, was particularly affected by deficient Ulk1 in terms of contractility (Figure 2). Fast-twitch fibers are characterized by less endogenous antioxidant protein content and there is a strong relationship between autophagy-related protein contents and muscle fiber type such that protein contents are greater in predominately slow-twitch muscle (e.g., soleus muscle) compared to predominately fast-twitch muscle (e.g., white vastus) [39,40,41]. Lifelong Ulk1 deficiency in the fast-twitch muscle fibers of MKO mice may have further limited the mitochondrial maintenance capacity via autophagy and it stands to reason then, that in this new cellular environment the fast-twitch fibers accumulated damaged mitochondria more quickly, produced more ROS as a consequence, and had less endogenous protection from ROS-induced damage [40,41]. The age-related preferential loss of fast-twitch fibers is multi-faceted beyond the regulation of autophagy alone, but it is reasonable to consider impaired autophagy throughout life as contributing to this age-related phenotype [42].

Age-related muscle weakness is associated with a reduction in motor units and some of the denervated muscle fibers can be inappropriately reinnervated such that a slow-twitch fiber is innervated by a fast-fatigable motor unit leading to altered recruitment patterns and changes in NMJ structure. Carnio et al., investigated the relationship between autophagy and NMJs specifically using ATG7 knockout mice and found that the muscle fibers from KO mice were expressing more NCAM, an attractant for alpha motoneurons, as a way to recruit new terminal axons to NMJs [7]. It is unclear the extent to which our results support the work of Carnio et al., as our aged autophagy deficient mice actually appeared to have greater innervation (Figure 4) [7]. NMJ structure and innervation are a dynamic, ongoing processes and there is potential that we captured ongoing reinnervation in the MKO mice subsequent to loss of innervation. Alternatively, the absence of Ulk1 could be associated with an undefined compensatory mechanism to increase motor neuron innervation in order to try and improve muscle contractile function (larger motor unit pools). Nonetheless, data presented here and reported by Carnio et al., agree that deficient autophagy results in altered NMJ structure and innervation ratios [7] and altering autophagy may be a therapeutic target to mitigate NMJ changes with age.

Throughout life there is ongoing myonuclei turnover in uninjured muscle fibers in order to maintain muscle homeostasis. Specifically, satellite cells are reported to be responsible for this myonuclei turnover and this process results in increased centrally-located nuclei with age, a marker typically reflecting muscle injury and ongoing repair [43,44,45]. It is unclear why centrally-located nuclei increase with age, but it appears to coincide with age-related changes in NMJ integrity [44,46]. Autophagy deficiency may pre-dispose fibers to NMJ remodeling with age, and this could influence satellite cell dynamics and/or a muscle fibers susceptibility to contraction-induced injury based on changing motor unit recruitment patterns. The inverse is also possible, as we reported a protracted recovery process after injury in autophagy deficiency muscle and this could influence satellite cell behavior and NMJ integrity [30]. Toward therapeutics, caloric restriction (a potent autophagy stimulus) decreases NMJ denervation and centrally located nuclei in aged mice [46]. Collectively, there is support for further investigating the role of autophagy in mediating the relationship between NMJ and myonuclear maintenance in aged muscle.

We acknowledge that the Ulk1 muscle-specific knockout mouse used herein does not perfectly model the natural aging changes in autophagy-related protein detected in human and mouse muscle (Figure 8). Specifically, our results actually indicated greater Ulk1 content in aged compared to young skeletal muscles. Aging is associated with a preferential loss of fast-twitch fibers, therefore the greater Ulk1 protein content detected may have resulted from a survivor bias, i.e., slow-twitch fibers make up a greater proportion of total fibers in aged muscle. Perhaps more important than total protein content, as a kinase Ulk1 has downstream molecular signaling responsibilities, particularly in initiating autophagosome assembly and potentially autophagy flux. In both human and mouse muscle, we detected a decrease in the activated-to-total Ulk1 ratio with age. The amount of activated Ulk1 necessary for basal autophagy and dynamic changes in autophagy (e.g., with exercise) is unclear and warrants further investigation across age and disease progression. Results from this study should be considered from the perspective that Ulk1 and its specific signaling capacities were diminished and may therefore exaggerate the natural role of Ulk1 in muscle aging. Alternatively, physiological results from this study may appear modest, considering the dramatic effects of other autophagy-related protein knockout studies [7]. Toward that end, it has been suggested that the Ulk2, a homologue of Ulk1 that shares 78% of its protein kinase domain, may be upregulated to compensate for the loss of Ulk1 [47]. Unfortunately, the ability of Ulk2 to compensate is not fully understood and appears to differ between different cell types 61. Investigations in young Ulk2 skeletal muscle-specific knockout mice have revealed greater ubiquitinated protein aggregates but without impairment in autophagy flux with a fasting stimulus, [48], which is a minor shift in contrast to the work reported herein (Figure 7). We did not measure Ulk2 to determine if there was a compensatory change in its protein content and considering the growing evidence of its contribution to muscle homeostasis it should be acknowledged that the presence of Ulk2 may have lessened the severity of our knockout model.

In summary, lifelong Ulk1 deficiency results in an accumulation of dysfunctional, ROS producing mitochondria that coincides with reduced muscle force production, denervation, and increased centrally-located nuclei. This aging phenotype may be due to a reduction in autophagosome degradation as a consequence of absent Ulk1 signaling. Considering that Ulk1 activation naturally declines with age and its dual role in autophagosome formation and degradation, Ulk1 may provide a potential therapeutic target to maintain muscle quality throughout life.

## 4. Materials and Methods

### 4.1. Ethical Approval and Animal Model

C57Bl/J6 Muscle-specific Ulk1 knock-out mice (MKO) with myogenin-Cre and LoxP flanked Ulk1 and their myogenin-Cre negative littermates (LM) were bred in-house and housed 5 per cage in a temperature-controlled facility with a 12:12 h light:dark cycle and aged to either 4 months, 16 months (middle-aged) or 22 months (old). All mice had ab libitum access to food and water throughout the duration of the experiment.

### 4.2. Experimental Design

This study was designed to assess aging muscle health and function in a model of lifelong autophagy deficiency. The first cohort of mice consisted of Ulk1 MKO (*n* = 10) and LM (*n* = 10) mice that underwent longitudinal tracking of body mass and in vivo muscle strength every two months beginning at 12 months of age. Mice were sacrificed at 22 months of age based on a greater than 80% survivorship rate of our colony and published estimates [49]. At sacrifice, muscles were harvested for in vitro muscle function analysis, mitochondrial function analysis, enzyme kinetics, and immunohistochemistry. An additional cohort of MKO and LM mice were aged to 4- and 16-months and evaluated in the following ways: (i) to determine age-related changes in mitochondrial remodeling proteins and total Ulk1 protein content and activation; and (ii) to determine the extent to which Ulk1 participates in autophagy flux using multi-photon microscopy. These earlier timepoint analyses can provide insight into the physiological changes detected in the aged mice. Aging is a condition that effects multiple limb skeletal muscles throughout the body. For practical reasons (i.e., insufficient muscle mass), not all tissues could be assayed for all variables (Table 1).

### 4.3. In Vivo Assessment of Muscle Function

Peak isometric torque of the ankle dorsiflexors (tibialis anterior (TA), extensor digitorum longus (EDL), and extensor hallucis longus 21 muscles) was assessed as previously described [50]. Briefly, mice were anesthetized using 1–3% isoflurane in oxygen, the left hind limb was shaved and aseptically prepared, and the foot was positioned into a foot-plate attached to the servomotor (Model 129 300C-LR; Aurora Scientific, Aurora, ON, Canada) where the ankle joint was adjusted to a 90° angle and secured at the knee joint. Platinum-Iridium (Pt-Ir) needle electrodes were inserted percutaneously on both sides of the peroneal nerve and the testing platform was maintained at 37 °C throughout the optimization and muscle stressor protocols. Optimal muscle stimulation was achieved by increasing the current in increments of 0.2 mAmps until a tetanic contraction was observed and muscle torque did not increase with further stimulation. Muscle torque (mN·m) was normalized to body mass (kg) to account for the large variability in body size, and later by TA muscle cross-sectional area (CSA). The force frequency relationship was assessed by measuring torque as a function of the following stimulation frequencies; 5, 10, 20, 40, 60, 80, 100, 150, and 200 Hz.

### 4.4. In Vitro Assessment of Muscle Function

Extensor digitorum longus (EDL) and soleus (SOL) muscles were excised and analyzed for force-generating capacities in vivo, as previously described [51,52,53]. Muscles were mounted on a dual-mode muscle lever system (300B-LR; Aurora Scientific Inc., Aurora, ON, Canada) in a 0.38-mL bath filled with Krebs Ringer bicarbonate that was maintained at 25 °C. Contractile characteristics tested included the following: peak twitch force, peak isometric tetanic force, time-to-peak twitch force and twitch half-relaxation time, and the maximal rates of tetanic contraction and relaxation.

### 4.5. Oxygen Consumption Rates

High resolution respirometry (Oroboros O2k) of permeabilized muscle fibers was used to assess mitochondrial oxygen consumption rates because permeabilized muscle fibers are a better representation of aging mitochondria physiology compared to isolated mitochondria [54]. Briefly, the medial portion of the gastrocnemius muscle was dissected into fiber bundles and permeabilized with saponin as previously described [30]. Mitochondrial leak respiration was accomplished by the addition of glutamate (10 mM), malate (5 mM), and succinate (10 mM). State III respiration was accomplished by adding ADP (5 mM) after leak respiration had been recorded. After state III respiration was reached, Cytochrome C (10 µM) was added to assess mitochondrial membrane quality to ensure no damage was obtained during the muscle fiber bundle dissection and permeabilization steps. Any test where a cytochrome C rate exceeded a 10% increase over the state III respiration rate was deemed as damaged and excluded from further analysis. Finally, FCCP (1uM) was added to obtain an uncoupled respiration rate. Baseline rates (the rate before any substrates are added) were subtracted out from all other rates and rates were normalized to wet weight of muscle fiber bundles added to chamber and citrate synthase values to account for differences in mitochondrial content.

### 4.6. ROS Production

ROS production was assessed by quantifying resoflourin (red fluorescence) produced from the reaction of H_2_O_2_ and Amplex UltraRed (AmR, 10 μM) catalyzed by horseradish peroxidase (HRP, 1 U/mL) during the oxygen consumption measurements. ROS rates were normalized by oxygen consumption rates during leak respiration to account for differences in total oxygen flux through the system.

### 4.7. Enzyme Assays

Citrate synthase enzyme activity was assessed to quantify mitochondrial content [55]. The lateral portion of the gastrocnemius muscle remaining after fiber dissection was homogenized in 33 mM phosphate buffer (pH 7.4) at a muscle to buffer ratio of 1:40 using a glass tissue grinder. Citrate Synthase activity was measured from the reduction of DTNB over time, as previously described [50].

### 4.8. Mitochondrial DNA Analysis

Relative mtDNA copy number analysis was performed by the Oklahoma Nathan Shock Center on Aging (Oklahoma City, OK, USA) by using the 2^−∆∆ct^ method. TERT was used as the nuclear reference and basepairs 257–557 and 14,927–15,227 were used for mtDNA as previously described [56] on DNA isolated from gastrocnemius muscle.

### 4.9. Immunohistology

The TA muscle was used to assess fiber type differences and cross-sectional area (CSA) of fibers. First, the TA muscle was cut cross-sectionally at 8 µm at the mid-belly of the muscle. Immunohistochemical staining of the TA muscle was conducted with a combination of MyHCSlow (BA-F8, mouse monoclonal IgG2b; 5 µg/mL), MyHCFast (F59, mouse monoclonal IgG1; 5 µg/mL), MyHC2A (SC-71, mouse monoclonal IgG1; 5 µg/mL), MyHC2B (BF-F3, mouse monoclonal IgM; 5 µg/mL), DAPI (Molecular Probes D21490, 1 µg/mL), and Wheat Germ Agglutinin (WGA, Molecular Probes W11262; 594-conjugated, 3 µg/mL). Second, the diaphragm muscle was cut longitudinally at 30 µm through the mid-costal region of the muscle and stained with combinations of α-bungarotoxin (Molecular Probes B13422; 488-conjugated, 1 µg/mL), neurofilament (2H3, mouse monoclonal IgG1; 0.5 µg/mL), and synaptic vesicle (SV2, mouse monoclonal IgG1; 2 µg/mL). Anti-BA-F8, F59, SC-71, BF-F3, 2H3, and SV2 were all obtained from the Developmental Studies Hybridoma Bank (Iowa City, IA, USA). For non-conjugated probes, appropriately paired secondary Alexa-Fluor conjugated antibodies at a dilution of 1:200 (Invitrogen (Carlsbad, CA, USA) A21123 or A21242; or Jackson ImmunoResearch Laboratories (West Grove, PA, USA) 115-005-020] were used. In all cases, the expected staining patterns in normal skeletal muscle were observed and the specificity of anti-labeling was confirmed by the absence of staining outside expected structures and was consistent with the manufacturer’s technical information.

Fluorescent Images were acquired at the University of Minnesota Imaging Center using a Nikon C2 automated upright laser scanning confocal microscope using 10x, 20x, or 40x Plan Apo objectives (NIR 0.45, 0.75, and 1.0 NA, working distance 4.0, 1.0 and 0.2 mm, respectively) with dual GaSP detectors (Nikon corporation, Minato City, Tokyo, Japan). Skeletal muscle fibers of the entire TA muscle were first classified singly as type I based on the expression of MyHCSlow and collectively as type II (type IIa, IIb, and IIx); less than 1% of all TA muscle fibers across groups were type I and were excluded from further analysis. Next, the entire TA muscle was used to quantify the number of centrally located nuclei (both single and multiple) and total fibers. Fiber type specific distributions and CSA was determined from representative images obtained systematically from three distinct regions of the TA muscle at the bone, middle, and superficial aspects; fibers were classified as type IIa, IIb, or IIx based on stating of MyHC2A, MyHC2B, or absence of staining respectively. FIJI (Bethesda, MD, USA) [57] was used to calculate the CSA and the proportion of fiber types. An average of 560 ± 124 fibers per animal were analyzed with no genotype difference for fiber type distribution at the distinct regions of the TA muscle (*p* = 0.5386), so fibers were averaged across animals.

The diaphragm muscle was used to assess NMJ integrity. Images of diaphragm muscle NMJs were obtained at a Z-step size of 1 µm to identify pre- and post-synaptic terminals. An average of 93 ± 39 en face NMJs per animal were classified as innervated or denervated based on complete or incomplete co-localization of the pre- and post-synaptic terminals, respectively. For display purposes only, images were down-converted, without introducing any changes in brightness or contrast and produced in Adobe Photoshop (Adobe Systems Inc., Mountain View, CA, USA). Laser intensity was consistent across imaging of the same probes. During all imaging and investigation, investigators were blinded to the experimental group.

### 4.10. Immunoblots

For autophagy-related protein content analysis, PVDF membranes were probed for the following proteins at a dilution of 1:1000: Ulk1 (RRID:AB 11178668), pUlk1 (s555) (RRID:AB_10707365), Dynamin-related protein Drp1 (D6C7) (RRID:AB 10950498), Pink1 (RRID:AB 11179069), Parkin (RRID:AB 10693040), BCL1 interacting protein BNIP3 (RRID:AB 2259284), and LC3B (RRID:AB 915950). Protein was extracted from the lateral portion of the gastrocnemius muscle. 30 μg of total protein was separated by SDS-PAGE, transferred onto a PVDF membrane, and immunoblotted as previously described [50]. Immunoblots were normalized to total protein in lane and quantified using Bio-Rad Laboratories Image Lab software (Hercules, CA, USA) [58,59,60].

### 4.11. Autophagy Flux Plasmid Transfection

Autophagy flux was assessed in young (4-month old) LM and MKO mice utilizing the in vivo expression of a GFP-LC3-RFP-LC3Δ plasmid imaged via 2-photon microscopy. GFP-LC3-RFP-LC3 plasmid (Addgene, Watertown, MA, USA, #84572) was prepared and transfected as previously described [30]. Briefly, 30 µg/µL of plasmid was injected into the TA and a BTX ECM 830 system equipped with 5 mm 2-needle arrays was used for electroporation. Muscles were imaged 4 weeks after transfection to allow for appropriate plasmid incorporation.

### 4.12. 2-Photon Scanning Microscopy and Image Analysis

A home-built microscope was used for multi-photon microscopy of transfected TA muscles [61]. Briefly, TA muscles were dissected into fiber bundles and stabilized on a dissection gel then imaged with the tissue immersed in Krebs Ringer buffer. We used a Ti:Sapphire Ultrafast laser with 940 nm excitation of GFP and 775 nm excitation of RFP (both 120 fs, linearly polarized). For GFP and RFP signal collection, we used 509/22 nm and 585/40 nm (Semrock) filters, respectively. Imaging was performed using a 60x water immersion objective lens (Olympus LUMFLN, Shinjuku City, Tokyo, Japan) with 1× PBS as immersion media. To process the images, we first denoised with 3D Gaussian blur filtering (standard deviation = 380 nm) and produced a binary mask from the RFP channel data using Otsu’s method. We then applied the same mask to both RFP and GFP channels, calculated the average fluorescence reading of the masked area in each, and finally the ratios of GFP to RFP for each stack (Videos S1 and S2).

### 4.13. Human Muscle Tissue Ethical Approval and Collection

Young (aged 20–40 y, *n* = 7) and old (aged 60–80 y, *n* = 7), male and female biopsy donors were recruited from Tompkins County, NY and the surrounding area. The following exclusion criteria were used: presence of a musculoskeletal disease, movement disorder, or other conditions known to influence skeletal muscle tissue (e.g., diabetes, cancer); high alcohol intake (>11 servings/wk for women, and >14 servings/wk for men); taking immunosuppressive or anti-coagulant medication; pregnant or breastfeeding; and recent weight changes or fluctuations. Biopsy tissue was collected following previously described methods [62], frozen in liquid nitrogen, and stored at −80 °C until processing.

### 4.14. Protein Isolation and Immunoblotting of Human Lysates

A 20–30 mg section of biopsy tissue was pulverized (Bessman pulverizer, Thermo Fisher Scientific, Waltham, MA, USA) and homogenized (VWR homogenizer) in lysis buffer (50 mM Tris, 150 mM NaCl, 1 mM EDTA, 1% NP-40, 0.5% Na-deoxycholate, 0.1% SDS, protease inhibitors (Roche, Basel, Switzerland), phosphatase inhibitors [Roche]). Lysates were incubated for 15 min on ice and centrifuged twice at 15,000× *g* for 20 min at 4 °C to remove insoluble material. Protein quantity was determined using a BCA assay (Thermo Scientific, Waltham, MA, USA). Lysates were then diluted in laemmli loading buffer, heated to 95 °C for 5 min, and separated on a 10% SDS gel (BioRad, Hercules, CA, USA). Proteins were transferred from the gel onto a PVDF membrane (Millipore, Burlington, MA, USA) at 30 V for 17 h at 4 °C.

### 4.15. Statistics

Changes in body mass, in vivo torque, and EC50 were analyzed by two-way repeated measures analysis of variance (ANOVA) with main effects of genotype and time. Additionally, two-way ANOVA was used to analyze fiber type distribution and CSA by fiber type with main effect of genotype. Differences in in vitro force production, NMJ innervation, mitochondrial function, mitochondrial content, ROS production, mtDNA damage, and autophagy protein expression between genotypes were analyzed by one-way ANOVAs with a main factor of genotype. Overall fiber CSA distribution was analyzed by Chi-squared. All data were required to pass normality (Shapiro-Wilk) and equal variance tests (Brown-Forsythe *F* test) before proceeding with the two-way repeated measures ANOVA. Significant interactions were tested with Tukey’s post hoc test using JMP statistical software v15 (SAS, Cary, NC, USA) to find differences between groups. Group main effects are reported where significant interactions were not observed. An α level of 0.05 was used for all analyses and all values are means ± SD unless otherwise noted.

## Figures and Tables

**Table 1:** Skeletal muscles collected postmortem, assays performed, and tissue function.

Tissue	Assay	Function
Extensor digitorum longus	In vitro contractile properties	Toe extension and foot dorsiflexion
Soleus	In vitro contractile properties	Foot plantarflexion
Tibialis anterior	In vivo contractile properties Autophagy flux (Chloroquine Treatment) Autophagy flux (multi-photon) MyHC isoform distribution (IHC) Fiber cross-sectional area (IHC) Fiber central nuclei (IHC)	Foot dorsiflexion
Gastrocnemius	Mitochondrial content (citrate synthase) Mitochondrial respiration mtDNA damage (PCR) Autophagy protein content (WB) Mitochondrial remodeling protein content (WB)	Foot plantarflexion
Diagram	Neuromuscular junction (IHC)	Ventilation

MyHC, myosin heavy chain; IHC, immunohistochemistry; WB, Western blot; PCR, polymerase chain reaction.

## Data Availability

All data are presented herein, or as Supplemental Materials, and may be requested from the authors.

## Supplementary Materials

The following are available online at https://www.mdpi.com/1422-0067/22/4/1937/s1, Figure S1: Muscle masses in old MKO and LM mice, Figure S2: In vivo torque normalized by CSA, Table S1: In vivo contractile properties, Table S2: In vitro contractile properties, Video S1: Multiphoton autophagy flux in LM mice, Video S2: Multiphoton autophagy flux in MKO mice.
