# Supplementary material for: Lifelong Ulk1-Mediated Autophagy Deficiency in Muscle Induces Mitochondrial Dysfunction and Contractile Weakness
Source: Int J Mol Sci. 2021 Feb 16;22(4):1937. doi: 10.3390/ijms22041937 (PMC7919824; doi:10.3390/ijms22041937)
Supplement: Supplementary file 1 [file ijms-22-01937-s001.zip › Supplemental Tables 1 and 2.pdf]

Supplemental Table 1: *in vivo* contractile properties

	LM (n=10) 12 Mo	LM (n=10) 24 Mo	MKO (n=10) 12 Mo	MKO (n=10) 24 Mo	P-value
TTP (ms)	58.8 (26.7)	71.1 (11.7)	71.1 (26.2)	82.8 (17.4)	0.960
HRT (ms)	47.1 (6.7)	47.7 (7.6)	48.1 (9.4)	46.9 (6.8)	0.489
+d*P*/d*t* (N/s)	330.5 (45.5)	291.6 (52.6)	288.9 (61.8)	285.7 (107.3)	0.523
-d*P*/d*t* (N/s)	288.0 (56.0)	277.4 (76.0)	271.9 (66.2)	244.3 (73.2)	0.667

Mean (SD) TTP=time-to-peak force. HRT=half-relaxation time. dP/dt=maximal rate of contraction. −dP/dt=maximal rate of relaxation

Supplemental Table 2: *in vitro* contractile properties

	LM (n=10)	MKO (n=10)	P-value
EDL			
Length (mm)	11.7 (0.5)	11.4 (0.4)	0.117
Mass (mg)	10.6 (1.2)	10.5 (1.8)	0.892
CSA	1.95 (0.27)	1.98 (0.35)	0.796
P_t (nM)	84.6 (14.6)	63.6 (14.8)	0.041
P_t TTP (ms)	20.4 (2.6)	18.5 (2.6)	0.166
P_t HRT (ms)	23.2 (3.1)	19.7 (3.9)	0.068
P_o +d*P*/d*t* (N/s)	14.5 (3.2)	13.2 (3.2)	0.453
P_o -d*P*/d*t* (N/s)	27.0 (5.1)	23.6 (7.5)	0.300
Soleus			
Length (mm)	10.1 (1.0)	10.1 (1.1)	0.995
Mass (mg)	9.0 (1.9)	10.0 (2.5)	0.442
CSA	1.21 (0.31)	1.33 (0.42)	0.511
P_t (nM)	20.8 (3.1)	17.1 (5.6)	0.475
P_t TTP (ms)	41.6 (10.9)	35.3 (9.6)	0.531
P_t HRT (ms)	61.5 (14.2)	47.8 (11.7)	0.277
P_o +d*P*/d*t* (N/s)	6.4 (1.6)	6.4 (2.5)	0.947
P_o -d*P*/d*t* (N/s)	5.3 (2.0)	6.9 (1.5)	0.405

Mean (SD). Pt=peak twitch force. TTP=time to-peak force. HRT=half-relaxation time. P_o=maximal isometric tetanic force. +dP/dt=maximal rate of contraction. −dP/dt=maximal rate of relaxation
