# Supplementary material for: Lifelong Ulk1-Mediated Autophagy Deficiency in Muscle Induces Mitochondrial Dysfunction and Contractile Weakness
Source: Int J Mol Sci. 2021 Feb 16;22(4):1937. doi: 10.3390/ijms22041937 (PMC7919824; doi:10.3390/ijms22041937)
Supplement: Supplementary file 1 [file ijms-22-01937-s001.zip › supplementary figures.pdf]

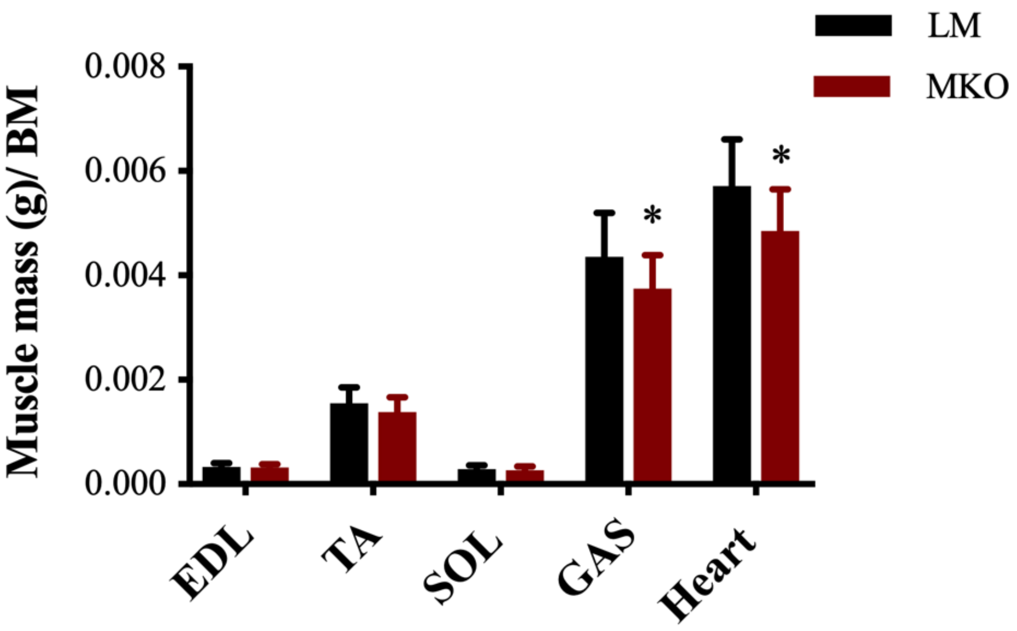

Supplemental Fig 1: Muscle masses in old MKO and LM mice. Muscle masses normalized to body mass. All data are presented as mean ± SD n=10 mice. *= significantly different from LM.

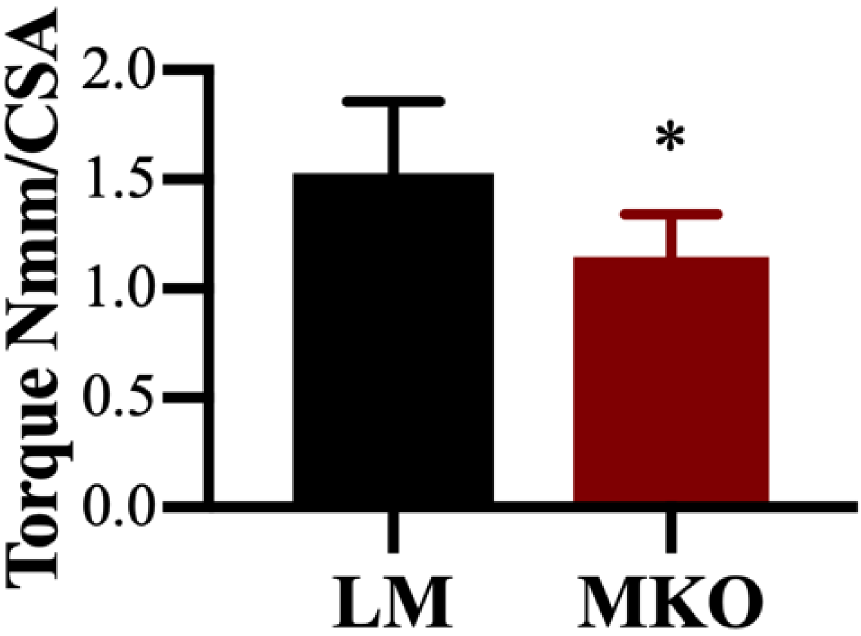

Supplemental Fig 2: *In vivo* torque normalized by CSA. *In vivo* torque normalized to CSA to account for fiber size differences between genotypes. All data are presented as mean ± SD n=10 mice. *= significantly different from LM.
